# Prognostic value of reactive hyperemia index using peripheral artery tonometry in patients with heart failure

**DOI:** 10.1038/s41598-023-27454-1

**Published:** 2023-01-04

**Authors:** Hack-Lyoung Kim, Woo-Hyun Lim, Jae-Bin Seo, Woo-Young Chung

**Affiliations:** Division of Cardiology, Department of Internal Medicine, Boramae Medical Center, Seoul National University College of Medicine, 20 Boramae-Ro 5-Gil, Dongjak-Gu, Seoul, 07061 Republic of Korea

**Keywords:** Cardiology, Medical research

## Abstract

Given the high prevalence and poor prognosis of heart failure (HF), finding prognostic factors for patients with HF is crucial. This study investigated the prognostic value of reactive hyperemia index (RHI), a measure of endothelial function, in HF. A total of 90 HF patients (mean age, 63.7 ± 13.2 years; female, 25.6%) with a history of hospitalization for HF treatment were prospectively enrolled. RHI was measured using digital arterial tonometry in a stable condition. Clinical events, including all-cause death and HF admission, were assessed. During the median follow-up of 3.66 years (interquartile range, 0.91–4.94 years), 26 clinical events (28.9%) occurred. Although there were no significant differences in risk factors and laboratory findings according to the occurrence of clinical events, the RHI value was significantly lower in patients with clinical events than in those without (1.21 ± 0.34 *vs*. 1.68 ± 0.48; *P* < 0.001). Kaplan–Meier survival analysis showed that a lower RHI value (< 1.48) was associated with a significantly higher incidence rate of clinical events (log-rank *P* < 0.001). In multivariable cox regression analysis, a low RHI value (< 1.48) was associated with an increased risk of clinical events (hazard ratio, 14.09; 95% confidence interval, 3.61–54.99; *P* < 0.001) even after controlling for potential confounders. Our study showed that reduced RHI was associated with an increased risk of adverse clinical outcomes in HF. This suggests that endothelial dysfunction may be an important prognostic marker in patients with HF.

## Introduction

Human endothelial cell (EC) secret nitric oxide, prostacyclin, and antithrombotic agents that dilate blood vessels and inhibit thrombus, inflammation and oxidative stress^1,2^. Therefore, EC function is crucial for protecting the vessel from atherosclerosis, degenerative change, and external injury^2^. Clinically, assessment of EC function is important because it can predict the development of future cardiovascular events^3,4^. Also, it has been reported that a therapeutic strategy to improve EC function is associated with improved cardiovascular prognosis^5^. The most widely used non-invasive method for measuring the function of an EC is flow-mediated dilation (FMD): estimation of endothelial function by measuring a change in diameter in antebrachial artery with blocking and loosening humeral artery flow^6^. Although FMD is non-invasive and has many data, it isn’t easy to clinically apply because it requires expensive equipment and technical skill for measurement and image analysis^7^. To overcome the disadvantages of FMD, the peripheral arterial tonometry (PAT) test newly attracted attention. It is a non-invasive, convenient measurement and does not require expensive tools, such as FMD, and proficiency due to the easy measurement method^8,9^. In addition, the value of reactive hyperemia index (RHI) measured by PAT had an excellent association with FMD value and efficacy in diverse clinical settings^10^.

Heart failure (HF) is highly prevalent with aging and an enormous burden on our society because of its morbidity and mortality^11,12^. Therefore, it is essential to find prognostic indicators of HF and use them for early detection and customized treatment for high-risk patients. Although several parameters of ventricular systolic^13^ or diastolic function^14^ and natriuretic peptide^15^ have been used as prognostic factors for HF patients, the mortality and readmission rates of HF are still high and have not improved significantly^11,12^. There is an unmet need for indicators that can be used to find HF patients at high risk.

In this respect, recent investigations on the relationship between the EC function and HF prognosis receive attention^16–19^. However, EC function was assessed by FMD in those studies. It would be beneficial if a method that can measure EC function more easily than FMD could predict HF-associated future clinical outcomes. This study aimed to investigate the prognostic value of RHI measured with PAT in patients with HF.

## Methods

### Study patients

This prospective study was performed in a general hospital in a big city (Seoul, Republic of Korea). Between June 2015 and May 2018, we recruited patients with HF at chronic and stable stages whose signs and symptoms have generally remained unchanged for at least one month. All patients had a prior history of hospitalization for HF, and HF was the primary diagnosis at the time of hospitalization. More specific inclusion criteria were as follows: (1) adult patients (≥ 18 years), (2) dyspnea with New York Heart Association class II or III, and (3) a history of hospitalization for HF within the last two years. Exclusion criteria were as follows: (1) resting dyspnea, (2) pulmonary edema, (3) hypotension (systolic blood pressure < 90 mmHg) or uncontrolled high blood pressure (systolic blood pressure < 180 mmHg), (4) uncontrolled arrhythmias, (5) difficulty in PAT measurement due to resting tremor or upper extremity blood vessel occlusion, (6) noncurative malignant tumors, (7) expected survival time shorter than one year due to underlying disease, and (8) pregnant and/or lactating women. The study was conducted in accordance with the principles established following the Helsinki Declaration, and the study protocol was reviewed and approved by the institutional review board (IRB) of Boramae Medical Center (Seoul, Republic of Korea) (IRB number 16-2015-41). Written informed consent was obtained from all study participants at the time of study enrollment.

### Data collection

Body mass index was calculated by dividing body weight (kg) divided by height squared (m^2^). Hypertension was defined based on the following criteria: (1) the previous diagnosis of hypertension by a physician, (2) current use of anti-hypertensive medications to control high blood pressure, or (3) systolic/diastolic blood pressure ≥ 140/90 mmHg on repeated measurements. Diabetes mellitus was diagnosed based on the following criteria: (1) the previous diagnosis of diabetes mellitus by a physician, (2) current use of anti-diabetic medications, or 3) fasting blood glucose level of ≥ 126 mg/dL from repeated measurements. Dyslipidemia was defined based on the following criteria: (1) the previous diagnosis of dyslipidemia by a physician, (2) current use of anti-dyslipidemic medications to control dyslipidemia, or (3) fasting blood level of low-density lipoprotein cholesterol ≥ 160 mg/dL. Patients who smoked regularly within a year are considered current smokers. Previous coronary artery disease was identified based on a history of myocardial infarction and coronary revascularization (percutaneous coronary intervention or coronary artery bypass surgery). The previous stroke was identified based on a history of acute onset of the neurologic deficit with documented brain lesions on imaging studies. After overnight fasting for about 12 h, venous blood in the antecubital vein was obtained, and the blood levels of the following parameters were analyzed: glucose, creatinine, total cholesterol, low-density lipoprotein cholesterol, high-density lipoprotein cholesterol, triglyceride, C-reactive protein and N-terminal-pro-brain natriuretic peptide (NT-proBNP). The glomerular filtration rate was calculated using the Modification of diet in renal disease (MDRD) study equation. Transthoracic echocardiography was performed, and the left ventricular (LV) ejection fraction was calculated using Simpson's biplane method. Data on septal E/e' and left atrial volume index was also obtained. Concomitant cardiovascular medications, including renin-angiotensin system blockers, beta-blockers, calcium-channel blockers, and statins, were identified.

### RHI

As previously described, EC function was measured using Endo-PAT2000 (Itamar Medical Ltd., Caesarea, Israel)^8,20,21^. The measurement was conducted in an independent space with a very quiet environment and constant temperature. On the day of the measurement, cigarette smoking or taking beverages containing caffeine was prohibited, and the drugs that were taken regularly were maintained. After resting for more than 15 min, a blood pressure cuff was wrapped around the upper arm, and a probe was placed on the finger. To occlude the brachial artery, a 50 mmHg higher pressure than systolic blood pressure was applied to the cuff. After maintaining the pressure for five minutes, the digital pulse amplitude was measured while rapidly releasing the upper arm cuff pressure. PAT signal from the contralateral finger was also measured as a control without applying pressure on the upper arm. RHI was defined as the ratio of pulse amplitude at baseline to one minute after deflation.

### Clinical event

The study's primary endpoint was a composite of net clinical events, including all-cause mortality and HF readmission.

### Statistical analysis

Numbers are expressed as mean ± SD or n (%). The characteristics of the two groups were compared using the Chi-square test and Student’s t-test for non-continuous and continuous variables, respectively. The cut-off value of RHI for the prediction of clinical events was obtained by the Youden index of receiver operating characteristic (ROC) curve analysis. Kaplan–Meier survival analysis was performed using the cut-off value of RHI, and the log-rank test was used to show statistical significance. The correlation between RHI and the number of rehospitalization was assessed using Spearman’s correlation analysis. Multivariable Cox regression analysis was performed to determine the independent association between RHI and the occurrence of clinical events. The following clinical covariates were controlled during the multivariable analysis: age, sex, body mass index, hypertension, diabetes mellitus, renal function, LV ejection fraction, and renin-angiotensin system blockers. All data were analyzed using the SPSS statistical package (IBM SPSS Statistics for Windows, Version 24; IBM Corp., Armonk, NY, USA). *P* value < 0.05 was considered statistically significant.

## Results

**Figure 1:**
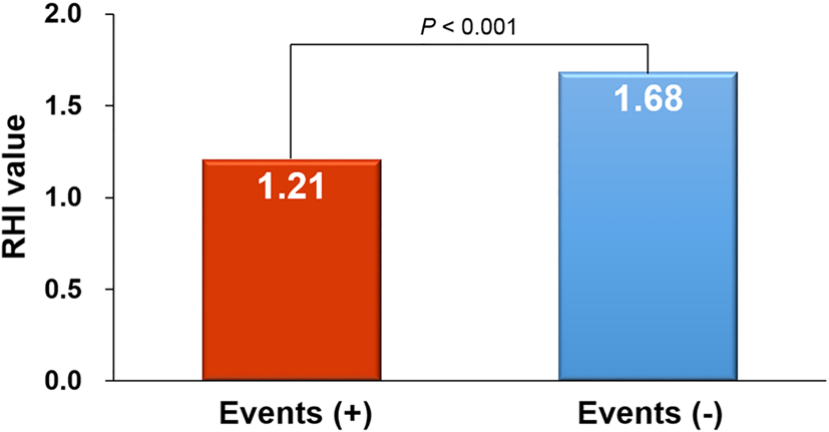
RHI according to the occurrence of clinical events RHI, reactive hyperemia index.

The mean age of the study patients was 63.7 ± 13.7 years, and 25.6% of patients were female. During the median follow-up duration of 3.18 years (interquartile range, 0.91–4.94 years), 26 patients (28.9%) suffered from clinical events. The baseline characteristics of study patients according to the occurrence of clinical events are shown in Table 1. There were no significant differences in age, sex, body mass index, cardiovascular risk factors, laboratory findings, and concomitant cardiovascular medications between patients with and without clinical events, except that the higher proportion of patients taking calcium channel blockers was higher in patients with clinical events than in those without (*P* = 0.045). The RHI value was significantly lower in patients with the clinical event than in those without (1.20 ± 0.34 *vs*. 1.68 ± 0.48; *P* < 0.001) (Fig. 1).

**Table 1:** Clinical characteristics according to clinical events.

Characteristic	Events ( +) (n = 26)	Events (−) (n = 64)	*P* value
Age, years	66.1 ± 15.3	62.8 ± 12.2	0.278
Female sex	6 (23.1)	17 (26.6)	0.731
Body mass index, kg/m^2^	23.9 ± 4.6	24.1 ± 3.9	0.858
**Cardiovascular risk factors**			
Hypertension	17 (65.4)	32 (50.0)	0.184
Diabetes mellitus	8 (30.8)	22 (34.4)	0.742
Dyslipidemia	5 (19.2)	25 (39.1)	0.070
Current smoking	10 (38.5)	22 (34.4)	0.714
Previous coronary artery disease	7 (26.9)	15 (23.4)	0.727
Previous stroke	4 (15.4)	7 (10.9)	0.559
**Laboratory findings**			
Fasting glucose, mg/dL	120 ± 53	119 ± 26	0.943
Estimated GFR, mL/min/1.73 m^2^	68.7 ± 29.7	73.2 ± 22.6	0.526
Total cholesterol, mg/dL	154 ± 31	156 ± 38	0.738
Low-density lipoprotein cholesterol, mg/dL	79.1 ± 16.5	96.4 ± 44.0	0.111
High-density lipoprotein cholesterol, mg/dL	44.3 ± 8.7	42.0 ± 10.5	0.400
Triglyceride, mg/dL	120 ± 73	126 ± 52	0.668
C-reactive protein, mg/dL	1.07 ± 1.78	0.89 ± 1.37	0.615
NT-pro-BNP, ng/mL	6906 ± 9625	4232 ± 6842	0.231
**Echocardiography results**			
Left ventricular ejection fraction, %	32.5 ± 13.8	33.3 ± 10.4	0.781
E/e'	17.7 ± 6.1	16.9 ± 8.2	0.669
Left atrial volume index, mL/m^2^	52.7 ± 16.5	48.9 ± 21.6	0.458
**Current medications**			
Beta blockers	21 (80.8)	53 (82.8)	0.818
Renin-angiotensin system blockers	22 (84.6)	56 (87.5)	0.715
Calcium channel blockers	14 (53.8)	20 (31.3)	0.045
Statins	14 (53.8)	46 (71.9)	0.100

Numbers are expressed as mean ± SD or n (%).

*GFR* glomerular filtration rate; *NT-pro-BNP* N-terminal-pro-brain natriuretic peptide.

**Figure 2:**
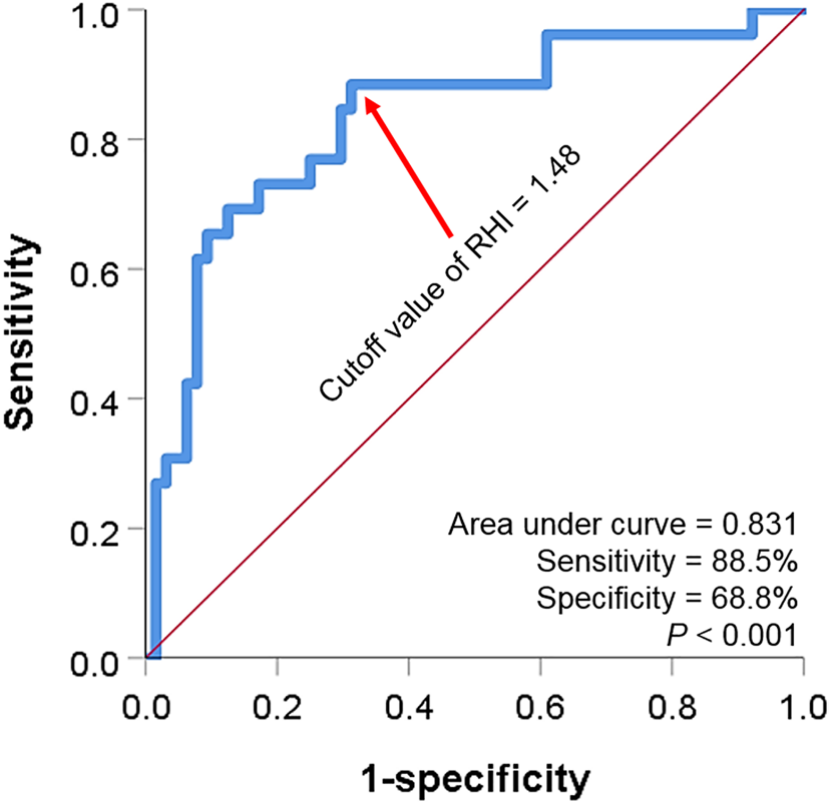
ROC curve analysis showing the cut-off value of RHI in the prediction of clinical events ROC, receiver operating characteristic; RHI, reactive hyperemia index.

ROC curve analysis showed that the cut-off value of RHI for the occurrence of clinical events was 1.48 (area under the curve, 0.831; sensitivity, 88.5%; specificity, 68.8%; *P* < 0.001) (Fig. 2). Based on the RHI 1.48, patients were divided into two groups, and their characteristics were compared (Table 2). There were no significant differences in most clinical findings except that renin-angiotensin system blockers were more commonly used in patients with higher RHI than those with lower RHI (*P* = 0.008).

**Table 2:** Clinical characteristics according to RHI value.

Characteristic	RHI < 1.48 (n = 43)	RHI ≥ 1.48 (n = 47)	*P* value
Age, years	64.7 ± 14.5	62.8 ± 11.8	0.498
Female sex	9 (20.9)	14 (29.8)	0.336
Body mass index, kg/m^2^	24.3 ± 4.5	23.8 ± 3.6	0.596
**Cardiovascular risk factors**			
Hypertension	26 (60.5)	23 (48.9)	0.273
Diabetes mellitus	12 (27.9)	18 (38.3)	0.296
Dyslipidemia	13 (30.2)	17 (36.2)	0.551
Current smoking	15 (34.9)	17 (36.2)	0.889
Previous coronary artery disease	9 (20.9)	13 (27.7)	0.458
Previous stroke	7 (16.3)	4 (8.5)	0.261
**Laboratory findings**			
Fasting glucose, mg/dL	116 ± 41	123 ± 32	0.489
Estimated glomerular filtration rate, mL/min/1.73m^2^	70.3 ± 26.5	73.7 ± 25.7	0.544
Total cholesterol, mg/dL	150 ± 27	160 ± 43	0.205
Low-density lipoprotein cholesterol, mg/dL	89.8 ± 34.9	94.0 ± 43.2	0.657
High-density lipoprotein cholesterol, mg/dL	42.5 ± 9.2	42.8 ± 10.9	0.887
Triglyceride, mg/dL	117 ± 49	131 ± 65	0.341
C-reactive protein, mg/dL	1.10 ± 1.83	0.80 ± 1.10	0.393
NT-pro-BNP, ng/mL	5,539 ± 8,187	4,693 ± 7,706	0.690
**Echocardiography results**			
Left ventricular ejection fraction, %	32.8 ± 11.6	33.3 ± 11.3	0.835
E/e'	18.6 ± 6.7	15.9 ± 8.3	0.135
Left atrial volume index, mL/m^2^	54.9 ± 21.5	45.5 ± 18.3	0.035
**Current medications**			
Beta-blockers	35 (81.4)	39 (83.0)	0.844
Renin-angiotensin system blockers	33 (76.7)	45 (95.7)	0.008
Calcium-channel blockers	20 (46.5)	14 (29.8)	0.102
Statins	28 (65.1)	32 (68.1)	0.765

Numbers are expressed as mean ± SD or n (%).

*MACE* major adverse cardiovascular event; *GFR* glomerular filtration rate; *NT-pro-BNP* N-terminal-pro-brain natriuretic peptide.

**Figure 3:**
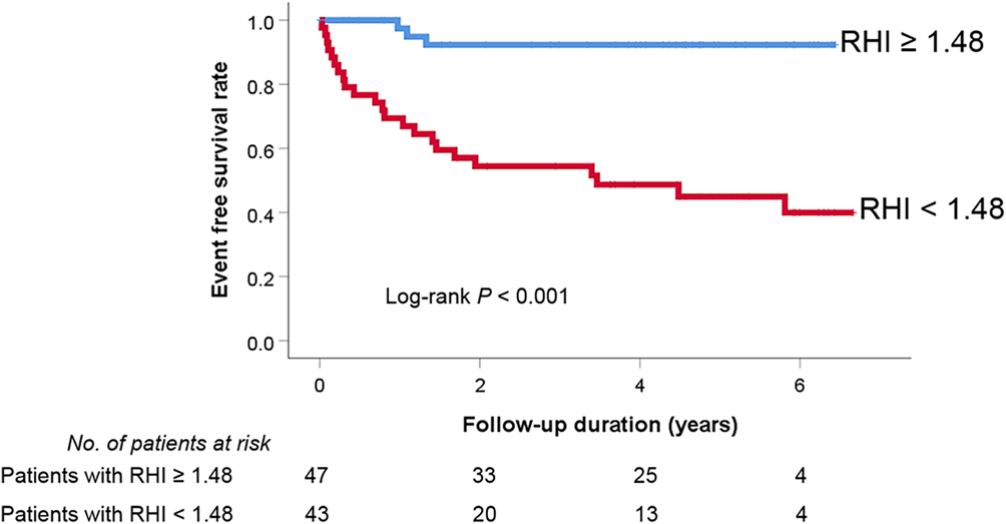
Kaplan–Meier survival curve showing the difference in even free survival rates according to RHI value ROC, receiver operating characteristic; RHI, reactive hyperemia index.

**Figure 4:**
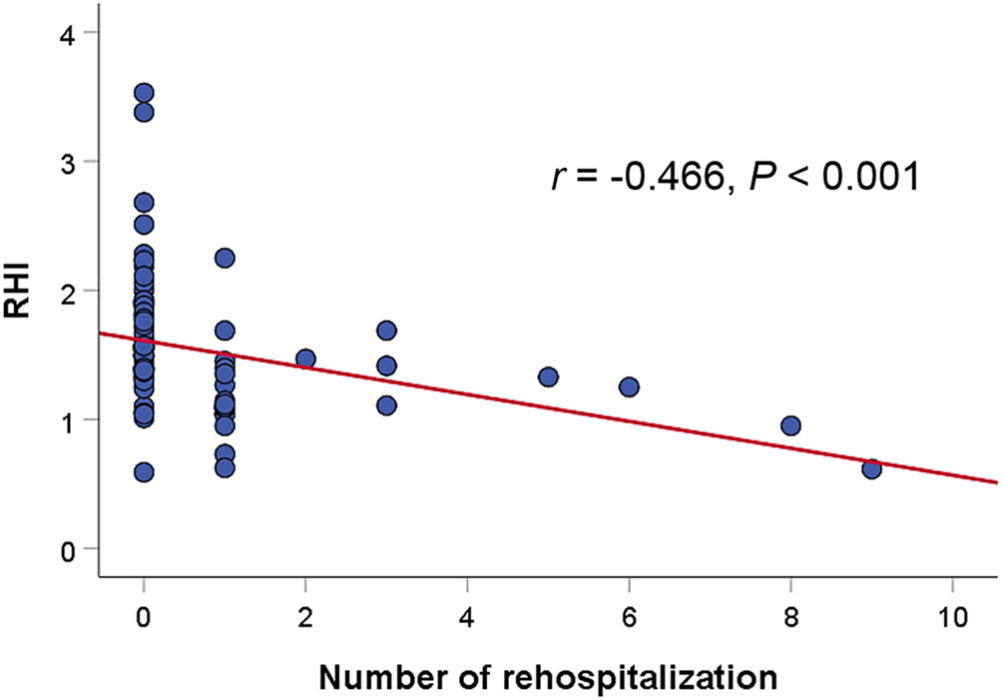
Scatter plots showing the association between RHI value and the number of rehospitalization RHI, reactive hyperemia index.

Kaplan–Meier survival curve analysis showed that the event-free survival rate was significantly lower in patients with lower RHI than in those with higher RHI (log-rank *P* < 0.001) (Fig. 3). There was a negative correlation between RHI value and the number of rehospitalization for HF treatment (r = −0.466; *P* < 0.001) (Fig. 4).

Multivariable cox regression analysis showed that lower RHI (< 1.48) was significantly associated with the occurrence of clinical events even after controlling for potential effects of several important clinical factors (hazard ratio, 14.09; 95% confidence interval, 3.61–54.99; *P* < 0.001) (Table 3).

**Table 3:** Multivariable analysis showing an independent association between RHI and occurrence of clinical events.

Variable	HR (95% CI)	*P* value
Age ≥ 65 years	2.06 (0.74–5.71)	0.162
Female sex	0.68 (0.23–2.05)	0.504
Body mass index ≥ 25 kg/m^2^	0.48 (0.18–1.27)	0.142
Hypertension	0.86 (0.27–2.72)	0.800
Diabetes mellitus	1.72 (0.63–4.69)	0.283
Estimated GFR < 60 mL/min/1.73 m^2^	1.44 (0.52–3.96)	0.479
Left ventricular ejection fraction < 50%	0.30 (0.05–1.62)	0.162
RAS blockers	1.83 (0.45–7.45)	0.396
RHI < 1.48	14.09 (3.61–54.99)	< 0.001

*RHI* reactive hyperemic index; *HR* hazard ratio; *CI* confidence interval; *GFR* glomerular filtration rate; *RAS* renin-angiotensin system.

## Discussion

The main findings of this study of HF patients are (1) baseline RHI was significantly lower in patients with clinical events than in those without, (2) a lower baseline RHI was associated with a higher risk of the occurrence of clinical events including death and HF readmission, and (3) RHI was negatively associated with the number of HF readmission.

Previous studies have evaluated the prognostic value of EC function in patients with HF^16–19,22–24^, (Table 4). However, in most studies, EC function was evaluated using FMD^16–19,22^. Heitzer et al.^22^ investigated 289 patients with depressed LV ejection fraction (35–50%), and demonstrated that reduced forearm vasodilatory response to acetylcholine was associated with worse clinical outcome. Another study of HF with reduced ejection fraction (HFrEF) (n = 149) found that a 1% decrease in FMD values increased the mortality risk by 20%^18^. In a study of 67 HF patients with both types of HF, Fischer et al.^17^ also showed the prognostic value of baseline FMD value predicting cardiovascular events during the clinical follow-up. Similarly, a more recent study of 82 HFrEF patients reported that the incidence of adverse cardiovascular events was significantly higher in patients with low baseline FMD^19^. Only a few studies have evaluated the association of endothelial function with prognosis in patients with HF using RHI^23,24^. In a Japanese study, baseline RHI was independently associated with cardiovascular events in 321 patients with HF with preserved ejection fraction (HFpEF) based on the finding that the risk of cardiovascular events increased by 20% with every 0.1 decreases in the RHI value^23^. Another Japanese study also showed that baseline low RHI value was an independent risk factor for HF-related death and rehospitalization in 159 HFpEF patients^24^. In line with these findings, our study showed that lower baseline RHI (< 1.48) was associated with a 14-fold increased risk of death and HF readmission in patients with HF.

**Table 4:** Summary of studies showing the prognostic value of endothelial function in patients with HF.

Source (year)	HF type	Number of study patients	Measure of endothelial function	Follow-up duration	Result	Summary of findings
Paine et al.^16^	HFrEF	156	FMD and hyperemic flow at brachial artery	5 years	+ /-	Reduced hyperemic flow, but not FMD, was associated with an increased risk of adverse events (aHR = 1.07)
Matsue et al.^24^	HFpEF	159	RHI	0.8 year	+	Log-transformed RHI was an independent predictor of HF-related events (aHR = 1.44 for a decrease of 0.1)
Akiyama et al.^23^	HFpEF	321	RHI	1.7 years	+	Baseline RHI was an independent predictor for cardiovascular events (aHR 1.20 for per 0.1 RHI decrease)
Shechter et al.^19^	HFrEF	82	FMD	1.2 years	+	Adverse cardiovascular events occurred more frequently in patients with low FMD (≤ 4.6%) than those with higher FMD (> 4.6%) (53.6% *vs*. 19.5%)
Heitzer et al.^22^	HFrEF	289	FMD	4.8 years	+	Blunted vasodilatory response to Ach was associated with adverse outcome (aHR = 1.06)
Katz et al.^18^	HFrEF	149	FMD	2.3 years	+	Reduced FMD in the brachial artery was associated with an increased mortality risk (aHR = 1.2 for 1% FMD decrease)
Fischer et al.^17^	HFrEF/HFpEF	67	FMD	1.9 years	+	Low FMD (< 6.2%) was an independent predictive factor for the occurrence of events (aHR = 1.33)

*HF* heart failure; *HFrEF* heart failure with reduced ejection fraction; *FMD* flow-mediated dilation; *aHR* adjusted hazard ratio; *HFpEF* heart failure with preserved ejection fraction; *RHI* reactive hyperemia index.

The exact mechanisms explaining the role of endothelial function in HF prognosis are not well elucidated. However, several hypotheses could be suggested. Many cardiovascular risk factors influencing HF prognosis also impair endothelial function^25–27^. Coronary artery disease is the main cause of HF and is also closely related to the prognosis of patients with HF^28^. A loss of endothelial nitric oxide synthase (eNOS) activity impairs coronary vasodilation, which leads to an aggravation of coronary artery disease^29^. In addition, dysregulation of nitric oxide contributes to the progression of HF directly affecting the myocardial contractile system and indirectly impairing myocardial perfusion^30,31^.

### Clinical implications

Even though the FMD test has the advantage of a non-invasive method for measuring the function of EC, it has difficulty in clinical application due to its expensive instrument, long learning time, being time-consuming for image analysis, and result influenced by sympathetic nervous system activity^7,24^. Considering these points, RHI, which can be measured more simply, may be an alternative to FMD in evaluating EC function. Compared to FMD, the main strength of PAT is operator-independent and easy to perform. PAT equipment is portable and requires little education and training to measure. Additionally, the machine automatically calculates the RHI value, eliminating the need for additional measurement and analysis by the inspector as with FMD. Therefore, PAT is more valuable than FMD for measuring endothelial function in mass screening^32^. A previous study reported that the value for EC function measured by RAT had a good correlation with the value measured by other tools such as FMD^33^. Moreover, a study analyzing Framingham cohort data has demonstrated that digital RHI had a stronger correlation with cardiovascular risk factors than FMD^34^. Therefore, RHI measurement may be applied to identify patients with HF at higher risk. We can improve patients’ prognoses by applying more intensive treatment to these high-risk HF patients. Renin-angiotensin system blockers and statins, expected to improve endothelial cell function, can be considered more actively as therapeutic agents in HF patients. Further studies are needed to confirm that the results of RHI-based management are beneficial and that reduction of RHI is related to improvement in HF prognosis.

### Study limitations

There are several limitations of this study. First, the number of analyzed patients was relatively small, and it was not possible to separately interpret the results according to HF types. For these reasons, it is presumed that the clinical characteristics were not significantly different according to the occurrence of clinical events or RHI. Second, because our study enrolled patients who had a history of HF hospitalization, its results cannot apply to all HF patients. Third, the fact that stable patients were enrolled in the study for accurate RHI measurement might cause selection bias. Fourth, we could not include some important HF medications in the study analysis. For example, only a small proportion of patients received spironolactone due to the small number of enrolled patients with HF with reduced ejection fraction. Additionally, sodium-glucose cotransporter-2 inhibitors and sacubitril/valsartan were unavailable in Korea as HF medications during the study period. Lastly, in our study, although RHI was identified as an important prognostic tool in patients with HF, it has not yet been validated. Further studies are warranted to confirm our results.

### Conclusions

Low RHI measured in patients with a history of hospitalization for HF was associated with worse clinical outcomes. This result provides additional evidence for the role of endothelial cell function as an important prognostic marker in patients with HF.

## Data Availability

All data generated or analyzed during this study are included in this article.

## Abbreviations

EC: Endothelial function
eNOS: Endothelial nitric oxide synthase
FMD: Flow-mediated dilation
HF: Heart failure
HFpEF: Heart failure with preserved ejection fraction
HFrEF: Heart failure with reduced ejection fraction
IRB: Institutional review board
LV: Left ventricular
MDRD: Modification of diet in renal disease
PAT: Peripheral arterial tonometry
RHI: Reactive hyperemia index
ROC: Receiver operating characteristic

## References

[1] Pober JS, Sessa WC (2007). Evolving functions of endothelial cells in inflammation. Nat. Rev. Immunol..

[2] Gao Y, Galis ZS (2021). Exploring the role of endothelial cell resilience in cardiovascular health and disease. Arterioscler. Thromb. Vasc. Biol..

[3] Perticone F (2001). Prognostic significance of endothelial dysfunction in hypertensive patients. Circulation.

[4] Rossi R, Nuzzo A, Origliani G, Modena MG (2008). Prognostic role of flow-mediated dilation and cardiac risk factors in post-menopausal women. J. Am. Coll. Cardiol..

[5] Flammer AJ, Luscher TF (2010). Human endothelial dysfunction: EDRFs. Pflugers Arch..

[6] Atkinson G, Batterham AM, Thijssen DH, Green DJ (2013). A new approach to improve the specificity of flow-mediated dilation for indicating endothelial function in cardiovascular research. J. Hypertens..

[7] Thijssen DHJ (2019). Expert consensus and evidence-based recommendations for the assessment of flow-mediated dilation in humans. Eur. Heart J..

[8] Bonetti PO (2004). Noninvasive identification of patients with early coronary atherosclerosis by assessment of digital reactive hyperemia. J. Am. Coll. Cardiol..

[9] Bonetti PO (2003). Enhanced external counterpulsation improves endothelial function in patients with symptomatic coronary artery disease. J. Am. Coll. Cardiol..

[10] Hamburg NM, Benjamin EJ (2009). Assessment of endothelial function using digital pulse amplitude tonometry. Trends Cardiovasc. Med..

[11] Park JJ (2021). Heart Failure Statistics in Korea, 2020: A report from the Korean society of heart failure. Int. J. Heart Fail..

[12] Bragazzi NL (2021). Burden of heart failure and underlying causes in 195 countries and territories from 1990 to 2017. Eur. J. Prev. Cardiol..

[13] Curtis JP (2003). The association of left ventricular ejection fraction, mortality, and cause of death in stable outpatients with heart failure. J. Am. Coll. Cardiol..

[14] Xie GY, Berk MR, Smith MD, Gurley JC, DeMaria AN (1994). Prognostic value of Doppler transmitral flow patterns in patients with congestive heart failure. J. Am. Coll. Cardiol..

[15] Januzzi JL (2006). NT-proBNP testing for diagnosis and short-term prognosis in acute destabilized heart failure: an international pooled analysis of 1256 patients: The International Collaborative of NT-proBNP Study. Eur. Heart J..

[16] Paine NJ (2016). Reactive hyperemia is associated with adverse clinical outcomes in heart failure. Am. Heart J..

[17] Fischer D (2005). Endothelial dysfunction in patients with chronic heart failure is independently associated with increased incidence of hospitalization, cardiac transplantation, or death. Eur. Heart J..

[18] Katz SD (2005). Vascular endothelial dysfunction and mortality risk in patients with chronic heart failure. Circulation.

[19] Shechter M, Matetzky S, Arad M, Feinberg MS, Freimark D (2009). Vascular endothelial function predicts mortality risk in patients with advanced ischaemic chronic heart failure. Eur. J. Heart Fail..

[20] Koo BK, Chung WY, Moon MK (2020). Peripheral arterial endothelial dysfunction predicts future cardiovascular events in diabetic patients with albuminuria: A prospective cohort study. Cardiovasc. Diabetol..

[21] Kang J (2018). Endothelial function estimated by digital reactive hyperemia in patients with atherosclerotic risk factors or coronary artery disease. Heart Vessels.

[22] Heitzer T, Baldus S, von Kodolitsch Y, Rudolph V, Meinertz T (2005). Systemic endothelial dysfunction as an early predictor of adverse outcome in heart failure. Arterioscler. Thromb. Vasc. Biol..

[23] Akiyama E (2012). Incremental prognostic significance of peripheral endothelial dysfunction in patients with heart failure with normal left ventricular ejection fraction. J. Am. Coll. Cardiol..

[24] Matsue Y (2013). Endothelial dysfunction measured by peripheral arterial tonometry predicts prognosis in patients with heart failure with preserved ejection fraction. Int. J. Cardiol..

[25] Coles AH (2015). Magnitude of and prognostic factors associated with 1-year mortality after hospital discharge for acute decompensated heart failure based on ejection fraction findings. J. Am. Heart Assoc..

[26] Choi DJ (2011). Characteristics, outcomes and predictors of long-term mortality for patients hospitalized for acute heart failure: A report from the korean heart failure registry. Korean Circ. J..

[27] Bonetti PO, Lerman LO, Lerman A (2003). Endothelial dysfunction: A marker of atherosclerotic risk. Arterioscler. Thromb. Vasc. Biol..

[28] Lala A, Desai AS (2014). The role of coronary artery disease in heart failure. Heart. Fail. Clin..

[29] Farah C, Michel LYM, Balligand JL (2018). Nitric oxide signalling in cardiovascular health and disease. Nat. Rev. Cardiol..

[30] Jones SP (2003). Endothelial nitric oxide synthase overexpression attenuates congestive heart failure in mice. Proc. Natl. Acad. Sci. U.S.A.

[31] Hare JM, Colucci WS (1995). Role of nitric oxide in the regulation of myocardial function. Prog. Cardiovasc. Dis..

[32] Rosenberry R, Michael DN (2020). Reactive hyperemia: A review of methods, mechanisms, and considerations. Am. J. Physiol. Regul. Integr. Comp. Physiol..

[33] Dhindsa M (2008). Interrelationships among noninvasive measures of postischemic macro- and microvascular reactivity. J. Appl. Physiol..

[34] Mitchell GF (2004). Local shear stress and brachial artery flow-mediated dilation: the Framingham Heart Study. Hypertension.

